# A Review of Corrosion in Aircraft Structures and Graphene-Based Sensors for Advanced Corrosion Monitoring

**DOI:** 10.3390/s21092908

**Published:** 2021-04-21

**Authors:** Lucy Li, Mounia Chakik, Ravi Prakash

**Affiliations:** 1 Aerospace Research Centre, National Research Council Canada, Ottawa, ON K1A 0R6, Canada; Lucy.Li@nrc-cnrc.gc.ca; 2 Department of Electronics Engineering, Carleton University, Ottawa, ON K1S 5B6, Canada; MouniaChakik@cmail.carleton.ca

**Keywords:** aircraft corrosion, cost of corrosion, corrosion environment and by-products, corrosion management, graphene derived materials, graphene-based sensors, thin-film chemical sensors, fiber optics

## Abstract

Corrosion is an ever-present phenomena of material deterioration that affects all metal structures. Timely and accurate detection of corrosion is required for structural maintenance and effective management of structural components during their life cycle. The usage of aircraft materials has been primarily driven by the need for lighter, stronger, and more robust metal alloys, rather than mitigation of corrosion. As such, the overall cost of corrosion management and aircraft downtime remains high. To illustrate, $5.67 billion or 23.6% of total sustainment costs was spent on aircraft corrosion management, as well as 14.1% of total NAD for the US Air Force aviation and missiles in the fiscal year of 2018. The ability to detect and monitor corrosion will allow for a more efficient and cost-effective corrosion management strategy, and will therefore, minimize maintenance costs and downtime, and to avoid unexpected failure associated with corrosion. Conventional and commercial efforts in corrosion detection on aircrafts have focused on visual and other field detection approaches which are time- and usage-based rather than condition-based; they are also less effective in cases where the corroded area is inaccessible (e.g., fuel tank) or hidden (rivets). The ability to target and detect specific corrosion by-products associated with the metals/metal alloys (chloride ions, fluoride ions, iron oxides, aluminum chlorides etc.), corrosion environment (pH, wetness, temperature), along with conventional approaches for physical detection of corrosion can provide early corrosion detection as well as enhanced reliability of corrosion detection. The paper summarizes the state-of-art of corrosion sensing and measurement technologies for schedule-based inspection or continuous monitoring of physical, environmental and chemical presence associated with corrosion. The challenges are reviewed with regards to current gaps of corrosion detection and the complex task of corrosion management of an aircraft, with a focused overview of the corrosion factors and corrosion forms that are pertinent to the aviation industry. A comprehensive overview of thin film sensing techniques for corrosion detection and monitoring on aircrafts are being conducted. Particular attention is paid to innovative new materials, especially graphene-derived thin film sensors which rely on their ability to be configured as a conductor, semiconductor, or a functionally sensitive layer that responds to corrosion factors. Several thin film sensors have been detailed in this review as highly suited candidates for detecting corrosion through direct sensing of corrosion by-products in conjunction with the aforementioned physical and environmental corrosion parameters. The ability to print/pattern these thin film materials directly onto specific aircraft components, or deposit them onto rigid and flexible sensor surfaces and interfaces (fibre optics, microelectrode structures) makes them highly suited for corrosion monitoring applications.

## 1. Introduction

Corrosion is the electrochemical deterioration of a metal because of its chemical reaction with a surrounding environment [1]. Metallic materials in aircraft structures, in particular aluminium and steel alloys, are susceptible to time-dependent effects of corrosion, which is often a slow process of material deterioration. A wide range of corrosion may occur in aircraft structures, such as general corrosion, pitting, stress-corrosion cracking, environmental embrittlement, and corrosion fatigue and exfoliation. Specific metallic materials are selected to fulfill aircraft design requirement based primarily on the performance attributes they exhibit, such as weight, stiffness, strength, electrical properties etc., rather than their ability to resist the onset of corrosion [2,3].

Corrosion prevention measures are commonly applied, such as surface treatments, corrosion-prohibiting primers, as well as protective coatings. However, such measures do not prevent corrosion completely, and regular inspection and maintenance are essential. Pollutants and moisture, direct attack from salt spray in marine environments or sulphate ions in urban environments, cleaning and de-icing fluids, create environments prone to corrosion in aircraft structures. Furthermore, erosion from sand and dust, impact from gravel, hail, dropped tools as well as fatigue loading could deteriorate protective layers, leading to direct exposure of metal alloys to in-service environments. The resistance of aircraft materials to corrosion can drastically change with only a small environmental change [1]. The challenge of corrosion prevention is further compounded by the environmental regulations that mandate the usage of chrome-free surface treatment and coating systems.

If undetected or left unaddressed, corrosion can affect structural integrity of an aircraft component by reducing material thickness and mechanical strength, or by accelerating fatigue crack development and stress corrosion cracking for the safety-of-flight structural components, ultimately jeopardizing the overall structural integrity [3,4]. In the most severe cases, corrosion can cause catastrophic structural failure. The 1988 incidence of the separation of a fuselage upper part on a Boeing 737 in service at Aloha Airlines was attributed to disbonding, corrosion and cracking problem [4]. Even though the maintenance record prior to the incidence showed that corrosion had been detected, the ongoing corrosion was accepted as a normal operating condition and no corrective actions had been undertaken [5]. The Aloha Airlines accident was the initiator of the recommendations to develop a corrosion prevention and control program that has since become a part of every operator’s maintenance programs for the aircraft.

The aim of this review is to provide a holistic overview of the existing challenges with regards to current gaps of corrosion detection and the complex task of corrosion management on an aircraft, with a specialized summarization of corrosion-related structural damages that are pertinent to the aviation industry, corrosion factors and graphene based novel optical, chemical and wireless sensors, which are highly applicable to near future implementation in advanced corrosion monitoring. Section 1, Section 2 and Section 3 of the manuscript is comprised of a comprehensive overview of the common forms of corrosion encountered in the aviation industry, and the associated inspection and maintenance costs. Section 4, Section 5 and Section 6 outline the in-service and environmental conditions conducive to corrosion in aircraft structures and the corresponding current detection methods and sensors including their strengths and shortcomings. Section 7 is a literature review of organic thin film materials implementation in corrosion monitoring with particular focus on graphene derived materials. The following sections describe emerging thin film sensing techniques of the corrosion environments and by-products highly suitable for aircraft health monitoring which are classified under three categories: fiber optics-based sensors (Section 8), thin film chemical sensors (Section 9) and wireless sensors (Section 10). Finally, the performances of the three techniques are evaluated and compared, and the challenges of implementing graphene derived materials in aircraft corrosion monitoring are discussed in the conclusion.

## 2. Forms of Corrosion Commonly Found on Aircrafts

**Figure 1:**
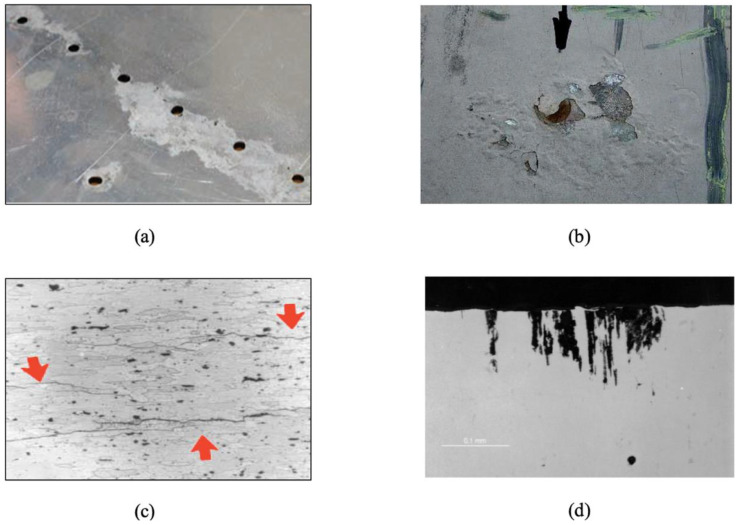
(**a**) Uniform corrosion on an aluminum aircraft structure ([used with permission from [6]). (**b**) Exfoliation corrosion at fastener holes in F-18 upper wing skins ([used with permission from [7]). (**c**) Intergranular corrosion of a failed 7075-T6 aluminum aircraft component ([used with permission from [7]). (**d**) Pitting corrosion resulted from the corrosion media attaching to the exposed end grains [used with permission from [8]).

Although corrosion on aircraft is considered to be nothing but rust of a metal part, corrosion comes in several different forms. Some of the common forms of corrosion—uniform, exfoliation, intergranular and pitting corrosion are shown Figure 1a–d, respectively. It can be seen that the different forms of corrosion often manifest themselves differently in appearance. They are often driven by different mechanisms, alone or synergistically. The effect of different corrosion forms on structural performance also varies significantly. The complexity and diversity of corrosion makes it challenging in corrosion monitoring and management.

An understanding of different corrosion forms, along with their mechanism, associated environment and impact on aircraft structures is the key to design and implementation of appropriate corrosion detection and measurement technologies. Table 1 provides a list of the corrosion forms on aircraft. It is worth noting that most structures are susceptible to one or more forms of corrosion.

## 3. Cost of Corrosion and Corrosion Management

With the mandate of an active corrosion prevention and control program and corrosion removal by the Federal Aviation Administration and military technical orders, catastrophic incidence and excessive downtime for structural repairs directly associated with corrosion has been largely avoided. Nevertheless, corrosion incurs a considerable impact on maintenance costs of an aircraft. The corrosion costs for all aviation and missiles of the United States Department of Defense were US $8.97 billion in the fiscal year of 2017, which increased to US $10.18 billion in financial year FY2018 [10,11]. For the US Air Force, the cost of corrosion in the FY2018 was $5.67 billion, accounting for 23.6% of total maintenance costs. Furthermore, corrosion also caused 89,653 NAD, about 14.1% of total NAD for the Air Force aviation and missiles. Another estimate of the corrosion costs conducted using a metric called the cost per day of availability (C/DA) indicates an estimated 4.8 day loss of availability for every weapon system of the U.S. Department of Defense (DoD), rendering over $20.6 billion or 20.7% of the total DoD maintenance expenditures for FY2016 [12]. The high corrosion management costs are not an issue for US Air Force only; the operators of all fixed- and rotary-wing aircraft with metallic structures and composite/metal hybrid structures face similar challenges.

As of today, corrosion management is based on “find and fix” philosophy. Corrosion inspections are routinely conducted using visual inspection, ultrasound, eddy current and other non-destructive inspections. These methods assess corrosion damage through mass loss, oxidation, and structural defects such as strain deformation, erosion, cracks and coating degradation [13]. Visual inspection lacks reliability since it fails to detect corrosion such as crevice corrosion in confined and hidden locations, small cracks and types of corrosions that start within the bulk of the material, such as pitting corrosion. Visual inspection is also impractical for aircraft bays with limited access, but these locations do experience external water ingress via changes in atmospheric pressure and temperature through joints and drainage holes. The current SBM (Scheduled Based Maintenance) are often not adequate in corrosion detection and monitoring, which is one of the main reasons behind the high cost of corrosion management. Other conventional monitoring techniques and devices, namely, corrosion coupons, electrical resistance and ultrasonic sensors suffer from limited sensing coverage and limited resolution and are prone to electronic issues requiring regular maintenance and replacement [13]. They may offer adequate assessment of corrosion rate and location, but do not provide any information about the type of corrosion nor its sources and conditions. In addition, the collected data can only be accessed during scheduled maintenance, which in some instances is well beyond prevention, requiring replacements of parts and resulting in extended downtime [13]. At the same time, the rejection criteria for corrosion on components and systems are defined on a case-by-case basis to ensure continued safe operation, once corrosion is found and reported. Lack of better tools for corrosion management is compensated by conservatism, leading to rejection of a corroded component even if the functionality of the component is not hampered [14].

There has been effort towards Condition Based Maintenance (CBM), or a holistic (cradle-to-grave) “damage and corrosion tolerance” management approach to reduce costs and ensure aircraft safety [8,15,16]. These maintenance methodologies are probabilistic-based prognostics and health management approach that utilize statistical tools such as failure modes and effects criticality analysis for reliability-centred maintenance [16]. The ability to detect and to monitor corrosion will allow for a more efficient and cost-effective corrosion management strategy by means of synchronization of corrosion removal with the maintenance plan to minimize maintenance costs and loss of availability [8]. These “anticipate and manage” approaches require on-board corrosion sensing systems to provide information regarding corrosion state and corrosion rates for corrosion prognostics, in order to mitigate safety risks, improve asset management, and reduce costs of aircraft maintenance. Another application of corrosion sensor is driven by environmental regulations mandating the replacement of hazardous, chrome-based primers with chrome-free alternatives on corrosion management of aircraft structures [17]. Corrosion sensors can provide data collected in the lab, on the ground, or on-board, to assess the performance of alternative paints and coating systems against corrosion for the specific flight envelop and in-service environments. Such data could offer the fleet-specific information based on aircraft usage for the development of a new maintenance strategy to ensure aircraft structural integrity and reduce maintenance cost.

Tremendous efforts have been made in the development of corrosion environmental sensors to detect the presence of corrosion, in quantifying corrosion rates with in-service environments and coating systems [18,19], as well as predicting fatigue life of aircraft structures through coupling or decoupling type corrosions, in particular pitting corrosion [20,21]. The impact of such developments on aircraft safety, availability and potential cost savings of aircraft sustainment via condition-based damage and corrosion management is huge, but there remain many challenges. First, corrosion is complex. While there are various mature corrosion sensors for detecting corrosion on the surface of a structure, it is difficult to detect corrosion that takes place underneath the surface, such as pitting and galvanic corrosion. Second, the Technology Readiness Level (TRL) of onboard corrosion sensing systems is not adequate for commercial applications. The onboard systems for corrosion monitoring are based on detection of corrosion environments such as temperature, humidity, time of wetness and conductance of electrolyte. High false-positive call rates are one of the key issues of such environmental corrosion sensors, which raises the question whether corrosion environment data alone is sufficient for reliable detection of corrosion. Qualification for on-board sensor sensitivity and durability is another key area to determine in-service failure rate and the service life of corrosion sensing systems. Third, there remains the challenge to integrate corrosion related information with aircraft conditioned based management systems. This requires not only the development of a corrosion prognostic system to quantify actual corrosion state based on sensor systems, but also structural fatigue life prediction tools to integrate corrosion as part of conditioned-based maintenance.

## 4. Corrosion Environments, By-Products and Corrosion Matrix

Aircraft structures are exposed to various in-service conditions that are conducive to corrosion. During take-off and landing, an aircraft will be impacted by dust, gravel, stones and de-icing salts from the runway, which can damage paints and expose bare metals. Similarly, aircraft is also exposed to operational and hazards such as oils, fluids and fuel, scratches and abrasions from maintenance actions, accidental damage during maintenance, battery acid, exhaust gases, toilet and galley spillages, and breakdown in seals and protective layers. In addition, maritime aircraft is exposed to severe salt spray, which is one of the most aggressive factors of corrosion [22]. A comprehensive corrosivity classification system ISO 9223 [23], created by the International Standards Organization (ISO), describes the combined impact of corrosivity variables on corrosion processes.

**Figure 2:**
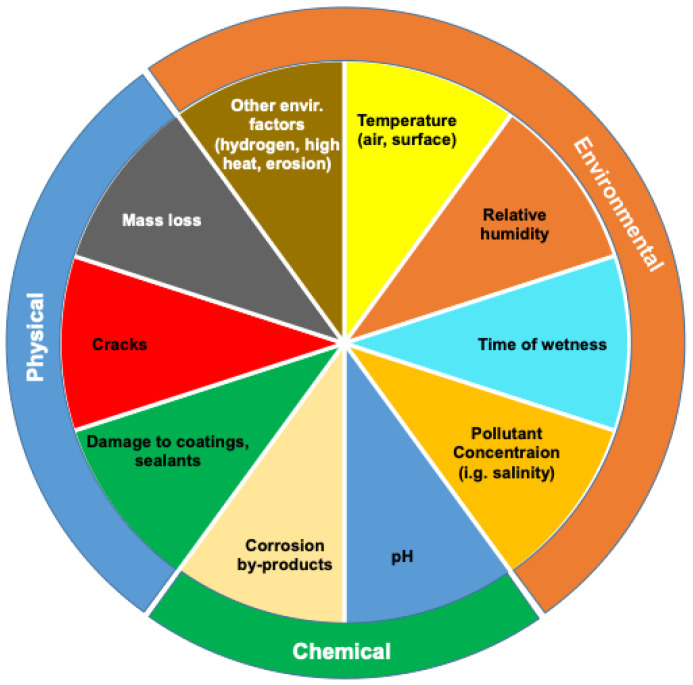
Corrosion matrix of physical, chemical and environmental factors.

Based on the fundamental mechanisms and characteristics of corrosion, a chart of corrosion matrix is proposed in Figure 2, which comprises of three categories of corrosion sensing and detection: physical, environmental, and chemical parameters. Environmental factors that have been found to influence corrosion include atmospheric pollutants, pH level, salt, temperature, humidity, and sand etc. The physical parameters of corrosion pertain to physical damage to protective coatings, paints, and sealants, and mass loss in the corroded metallic structure, and cracks of metallic structures that may be associated with corrosion. The physical indications of corrosion are often detected by non-destructive inspection (NDI) methods or structural health monitoring (SHM) technologies. NDI methods for corrosion include visual inspection, enhanced visual/optical NDI, ultrasonic, eddy current, thermography, radiography and other NDI methods [18], and SHM includes acoustic emission and fibre optics sensors among others [24]. It is worth noting that detection of damage of the protective coating may be categorized as an environmental factor for corrosion.

Corrosion environmental sensors do not detect corrosion directly, but they offer information to correlate to the location, time and rates of a corrosion event. A range of environmental parameters such as temperature, relative humidity, time of wetness, pollutant concentration among others have been found to be the key environmental factors associated with corrosion. Among the environmental factors, the most important environment of corrosion is the presence of electrolyte, which comprises of moisture and pollutants that can form on metallic surface. Moisture may be present in the form of rain, dew, condensation, or high humidity while pollutants typically refer to sea salts, acid sulfates, and acid chlorides that are diffused into moisture, forming an electrically conducting and chemically reactive solution. There are other types of atmospheric pollutants such as sulfur dioxide (SO_2_), sulfur trioxide (SO_3_), hydrogen sulfide (H_2_S), ammonia (NH_3_), chloride, smoke particles [22]; their concentration varies depending on the terrestrial location, season, and weather conditions, and other events such as volcanic eruption. Common electrolytes, especially salt (which is regularly seen in marine and coastal environments) is a key driver of corrosion and must be monitored for corrosion management. When salt infiltrates an area of wetness, not only does it promote electrical conductivity, but the chloride ions also prevent the regeneration of protective oxide layers on certain metals, thus inhibiting the metal from using any sort of protection against corrosion. The type of solution formed on the surfaces of metals, along with environmental conditions such as temperature, directly influences electrical conductivity and the rate of corrosion [25]. In the instances in which crevices exist and water becomes entrapped in a stagnant state, this can create a higher concentration of metal ions and thus create an electrical potential that can promote the onset of corrosion [26]. In presence of such high concentration of corrosive pollutants, metal ions may also exist on the surface of a structure, especially under conditions of alternate wetting and drying [5]. As a result, the time of wetness (TOW), which refers to the period of time during which the atmospheric conditions are favorable for the formation of a surface layer of moisture on a metal or alloy (or the duration of the electrochemical corrosion processes), is a key indicator of the corrosion rate [17,27,28]. TOW is one of the main factors in predicting the initiation and growth of corrosion. Detailed and science-based models are required to determine the historical corrosion environment that use meteorological and pollutant deposition data, along with environmental sensor data, and a suite of models that synergistically use this data to determine the historical corrosion environment. The corrosion environment can then be used with surface science and electrochemical models to predict the occurrence and severity of corrosion [29].

Temperature is also critical to the corrosion process. It does not just promote corrosion itself; it can also change the form of corrosion attack. For example, when temperature exceeds a certain threshold, corrosion can change from uniform to pitting corrosion, a more severe form of corrosion. Other factors such as change in pH, reduction in part thickness or mass loss, as well as damage to protective coatings can also be used to evaluate corrosion and corrosion rate. Corrosion by-products such as hydrogen and other chemicals, produced as a result of chemical reaction of metals and electrolyte is a direct indication of corrosion.

The ability to target and detect specific corrosion by-products associated with the metals being used on aircrafts, along with corrosion environment, can provide early corrosion detection as well as enhanced reliability of corrosion detection, which could lead to an improved aircraft corrosion management strategy to prevent structural failure, reduce corrosion management costs, and increase aircraft availability.

## 5. State-of-the-Art Corrosion Sensors

Proactive and effective corrosion management systems are built upon continuous on-asset corrosion sensing and monitoring. This can be achieved through measurements of physical, environmental and chemical corrosion parameters as shown in Figure 2, such as air temperature, surface temperature, relatively humidity, time-of-wetness, salt contamination, the accumulation of corrosion by-products, monitoring changes in appearance and part thickness, changes in pH, electrochemical characteristics, etc. [30,31]. Reliable corrosion sensing and monitoring for aircraft management often requires complimentary data in three categories: direct measurement of physical corrosion effects, measurement of environmental parameters, and measurement of chemical corrosion by-products. This dataset can be applied to obtain corrosion environment, corrosion severity and rates, which could then be integrated into aircraft damage tolerance model for condition-based management of a structure, or a digital twin that is a digital representation of an asset to enable better understanding and prediction of the performance of an aircraft.

The direct measurement of corrosion effects tracks changes directly correlated to the corrosion of a structure of interest. An example of this approach is to use electrical resistance probes. When materials corrode and begin to decrease in thickness, an increase in electrical resistance occurs, and this ramp in electrical resistance can be correlated to the onset of corrosion. Environmental sensors can be employed for humidity sensing, pH changes, surface temperature, salt contamination etc., to assess the severity of corrosion environment and to predict the corrosion rate of a structure. Lastly, measurement of corrosion by-products entails the usage of chemical sensors to target specific corrosion by-products or ions that are produced in chemical reactions of corrosion. For example, a sensor specifically designed to monitor the production of iron oxide or rust can be used to detect the onset of corrosion of iron [11]. Table 2 highlights common sensor types/methodologies that have been explored to achieve corrosion detection.

## 6. Current Field and On-Board Corrosion Monitoring Approaches

Currently, aircraft corrosion monitoring is based on “find and fix” protocols, focused on the physical corrosion parameters outlined in the corrosion matrix in Figure 2. These protocols rely on either visual inspection, field sensors or on-board sensors. Visual inspection often fails to detect early stages of corrosion damage, corrosion in locations with limited access as well as corrosion that is visually undetectable. Field sensors are used to monitor the corrosion rate and progression of aircraft structures by recreating flight conditions on the ground. However, data obtained on the ground may not accurately reflect the real conditions of corrosion events and progression during flights. Thus, field sensors are useful to develop tools for corrosion detection and monitoring with integration of the ground environmental data, and they are often suited for on-board applications as well.

On-board sensors, on the other hand, are sensors installed on aircraft structures that collect data on a continuous basis. There are several aircraft structural health monitoring (SHM) sensors currently in use for physical parameters (mass loss and cracks) and environmental conditions (relative humidity and temperature) monitoring. Physical parameters monitoring schemes are based on non-destructive inspection (NDI) techniques, such as, eddy current as in the case of the Meandering Winding Magnetometer developed by JENTEK [46], electrical resistance such as AirCorr O designed by the French Institute of Corrosion [47], and magnetic flux leakage for corrosion induced mass loss measurements developed by Battelle [48]. Current corrosion environments sensors focus on monitoring air temperature and relative humidity. These sensors include fiber optics temperature sensors developed by Luna [49] and air temperature and relative humidity sensor nodes and capacitive sensors developed by Luna and Senserion, respectively [50,51].

While Current aircraft structural health monitoring approaches may provide adequate measurements of physical and some environmental conditions, they have limitations and fail to address all the corrosion factors outlined in Figure 2. Inspection of physical damage is the most commonly used approach for corrosion detection, but detected damage may not necessarily be associated with corrosion. For example, liquid penetrant, eddy current and ultrasounds can detect defects in a material, but they do not distinguish between cracks and corrosion and the identification of defects is left up to operator experience and/or additional inspection methods [32]. Previous studies have demonstrated that the mass loss and cracks due to corrosion could be attributed to various forms of corrosion such as pitting and intergranular corrosion, and stress corrosion etc. [13,52]. Monitoring of physical parameters alone does not provide useful information about the form of corrosion, or its conditions and causes. Monitoring of relative humidity, air temperature and other environmental parameters demonstrates the potential for corrosion to occur but fails to detect corrosion events. Therefore, effective aircraft corrosion management should include preventive and early on monitoring focused on environmental factors specific to corrosion, particularly pollutants such as acidity and salinity, and corrosion by-products, namely, pH and metal ions [28,30]. Without direct detection of critical environmental parameters and corrosion by-products, inaccurate corrosion monitoring and associated high maintenance costs as well as prolonged down-times are expected.

Reliable corrosion monitoring requires comprehensive understanding of corrosion processes and a panoply of sensing mechanisms that offer both direct detection of corrosion events, and continuous measurement of environmental parameters pertaining to corrosion. An integrated sensing system can offer early on, reliable and real-time information about the structural health of aircrafts by providing information about the form, location and rate of corrosion. This assessment can be achieved by monitoring parameters that preclude or are responsible for corrosion, namely, environmental conditions including temperature, moisture, and acidity, as well as stress conditions resulting from sustained and cyclic loading. The assessment also encompasses corrosion by-products, including pH variations and metal ions, that are emblematic of early-on corrosion events [53]. Such methods rely on the use of stand-alone and distributed systems as well as wired and wireless sensing networks that can easily be embedded and integrated in structures and offer real-time detection of chemical species responsible and indicative of corrosion, and structural deformation or defects [53]. Such systems may be used to monitor a wide range of parameters simultaneously, using different techniques and different sensing materials. The generated data may be collected and processed by a common processing unit, minimizing thereby, the complexity of the network and the associated costs [54].

The discovery of organic semiconductors along with advancements in patterning and functionalization techniques have promoted significant innovation in sensor technology applicable in corrosion monitoring [55]. Organic thin film materials possess unique structural, electronic and chemical features that are ideal for the fabrication of state-of-the art sensors [55]. They have been the focus of vigorous research in chemical sensing, bio sensing and optoelectronics due to their high sensitivity associated with high surface area-to-volume ratio and their tunable selectivity [55,56]. Their promising physical properties allow for miniaturization of devices while maintaining high performance and set the ground for the development of flexible and wearable devices [55]. Organic thin film-based sensors are novel candidates to be used in corrosion monitoring which can benefit from their high sensitivity, selectivity, small footprint, compatibility to existing circuits and low power consumption.

The key challenge of chemical sensors for corrosion detection is the slow process of corrosion and extremely minute amount of corrosion by-products. No commercial products are available at the moment to offer detection that is sensitive and reliable enough to detect a range of chemical by-products of corrosion processes. This gap could be addressed by thin film material-based sensors for the application of high-fidelity, real-time corrosion detection and measurements. In the following section, the potential of organic thin film materials, specifically graphene, is discussed for the application of chemical, as well as environmental sensors for corrosion. Emerging sensing systems suitable for aircraft structural health monitoring are introduced, including optical fibers, chemical sensors, and wireless sensors. Examples of successful implementations of graphene in each approach as reported in literature are also discussed.

## 7. Implementation of Organic Thin Film Materials in Corrosion Sensing

Organic thin film conductors and semiconductors have attracted significant interest in electronic devices fabrication due to their abundance, high sensitivity, solution processability, mechanical flexibility and low-cost fabrication [56]. These properties, combined with scientific progress, led the way to significant improvements in the performance of organic semiconductor-based electronics, which made them comparable to their inorganic counterparts in term of carrier mobility and generated photocurrent [56]. The success of such devices is also due to improvements in fabrication techniques such as deposition conditions and growth rate optimization, in addition to the diversity of organic materials and the versatility of their electronic and optical functionalities [57].

Graphene has attracted great attention among available organic semiconducting materials. Its morphological characteristics, especially its high surface to volume ratio has been attractive for real-time sensing of biomolecules, gas and metals [58,59]. Graphene contains high electronic charge mobility, Young’s modulus, and thermal conductivity, which makes it an interesting candidate material for integrated circuitry, energy storage, and chemical and bio-electronic sensors. It is characterized by its high responsivity, large linear dynamic range (LDR), broadband absorption and field effect tunability [57]. It can be chemically or structurally modified to target specific molecules or ions through chemical functionalization as well as the introduction of nanopores [59].

The performance of graphene-based sensors depends strongly on the synthesis techniques which determine the purity, crystal structure and mechanical and electronic properties. Graphene and its derivatives can be synthesized using various chemical or mechanical processes which are broadly classified in two categories, top-down and bottom-up techniques [60]. The top-down techniques essentially use chemical/mechanical processes to produce graphene and graphene derivative materials from the naturally existing graphite mineral. This includes exfoliation (mechanical and liquid phase) for pristine graphene synthesis, oxidative exfoliation-reduction which produces graphene oxide (GO) and reduced graphene oxide (rGO) and unzipping of carbon nanotubes (CNTs) which results in single, bi- layer graphene (BLG) and few layers of graphene (FLG) [60]. In comparison, bottom-up techniques rely on solid carbon and carbon gas sources to chemically synthesize pristine and doped graphene on a substrate under controlled high temperature and pressure environment. These methods comprise of chemical vapor deposition (CVD), epitaxial grown and pulsed laser deposition (PLD) [61]. Presently, CVD and PLD produce the purest form of pristine graphene and graphene derived materials, but at higher cost than bottom-up techniques which are capable of large scale and relatively low-cost synthesis at the expanse of material quality in terms of electronic and mechanical properties [60].

Chemical functionalization of graphene is essentially performed by introducing a different species to graphene to enhance its electronic and optical properties as well as selective sensitivity [57]. For instance, GO is a functionalized form of graphene resulting from attaching oxygen atoms [57]. During the oxidation process of graphene, sp^3^ bonding is heavily interrupted and makes electrical conductivity of graphene oxide decrease drastically compared to pristine graphene [62]. GO can be modified to produce functionalized GO (FGO), which has an altered electronic conductivity (lower or higher depend on the functionalization type) compared to pristine graphene and GO [63,64]. Another benefit of GO compared to other organic thin film materials, is that it can be produced using cost-effective chemical methods, and in high yields from the inexpensive graphite raw material. In addition, due to its hydrophilic nature, stable aqueous colloids can be formed to facilitate the assembly of macroscopic structures by simple, low-cost solution deposition processes [65,66]. GO can be partly reduced to graphene-like sheets by removing the oxygen-containing groups with the recovery of a conjugated structure [66,67]. Reducing graphene oxide by fluorination has its own advantages as well. Owing to the low polarity of C–F bonds and the high polarity of oxygenic groups, characteristics of FGO could be tuned by controlling the C-F and C-O ratios. Oxygen or fluorine atom concentration can be tuned for different end-product usage [68]. The main advantage of FGO for sensing is its high electrocatalytic activity (augmented electron transfer), and specificity toward certain analytes due to the presence of C–F bonds [69,70]. It has been observed that low fluorinated graphene exhibits excellent sensing properties for a range of studied biomolecules and the low fluoride (F) content that augments the electron transfer from electrode to biomolecule (or vice versa) will neither significantly affect the electrical conductivity nor the wetting properties of graphene [71,72].

Aircraft corrosion monitoring techniques can benefit from the selective sensitivity of functionalized graphene by including graphene membranes sensitive to specific ions, molecules and environmental parameters. For instance, GO is sensitive to moisture and has been used in a variety of sensors like capacitive sensors, surface acoustic wave and RFIDs -based sensors [73,74,75]. rGO has been reported as a well-balanced temperature sensor, exhibiting good sensitivity, repeatability and environmental stability [76]. Graphene can also be used as the sensing material in ion-selective field effect transistors (ISFET) to measure the content of metal ions at low concentrations. Alves et al. proposed a graphene-based metal ion (Na, Co, Al, Cu) sensor in which graphene was functionalized with L-phenylalanine that attracts and bonds with metal ions. The sensor demonstrates high sensitivity to a variety of metal ions and shows real-time response [77]. Pristine graphene on the other hand, is highly reactive to oxygen carrying groups like OH^−^ and has efficient quenching capabilities making it suitable for both chemical and optical based pH sensing [44].

Graphene nanopores and nanochannels are other interesting products of graphene functionalization via the introduction of defects or pores in graphene membranes to enhance the selective sensitivity toward specific ions or molecules [78]. Here, the nanopores and nanochannels act as filters that allows the passage of some ions and restrict the passage of others [78,79]. To illustrate, Sint et al. explored the selective transport in graphene membranes by chemically modifying the pores and nanochannels to bond with specific ions and concluded that Nitrogen-ending and fluorine-ending functionalized pore are selective toward Li^+^, Na^+^ and K^+^, and hydrogen-ending pores are selective toward F^−^, Cl^−^ and Br^−^ [78]. Although experimental results showed that ion selective graphene nanopores exhibit good selectivity and excellent sensitivity, this technology is still at its infancy and requires further understanding of ion transport and interaction mechanisms in nanoconfined environments, in addition to overcoming challenges associated with nanopores and nanochannels fabrication and functionalization [78].

With the advent of such highly functionable and stable graphene derived materials, their integration into existing optical, electronic, chemical, and electrochemical detection systems has been vastly explored. The marriage of such uniquely tailored materials, with established detection systems, has yielded several highly promising recent outcomes of detecting metal ions, non-metal ions, metallic compounds, and environmental parameters, which are very relevant to aircraft corrosion monitoring. In the next sections, we have carefully selected research outcomes from recent publications that fit such device considerations and can therefore become suitable components of next-generation aircraft-based corrosion monitoring systems.

## 8. Fiber Optic Based Sensors

During the past few decades, optical fibers have drawn worldwide attention due to their larger bandwidth and long transmission ranges resulting from low power losses. Silica optical fibers are suitable for harsh environments due to their resistance to high temperature, corrosive environments and electromagnetic interference. Fiber optic-based sensors have emerged as promising structural health monitoring devices compared to their electronic counterparts, owing to their high sensitivity to temperature, pressure, mechanical stress and strain as well as acoustic vibrations [80]. Initially, optical fibers were implemented as point-wise sensors, which detect environmental parameters from a single individual location along the fibers [80]. The realization of various multiplexing schemes such as, wavelength-division, carrier frequency-division, time-division and spatial division multiplexing, allowed for the deployment of distributed fiber optic sensors that provide multiple point remote measurements using a common source and detection systems coupled with an array of fiber optic sensors. While this arrangement increases the complexity of the interrogation and the signal processing scheme, multiplexing enhances the quality of the measurements and reduces the costs associated with increasing the number of point-wise optical fibers [80]. In aircraft structural health monitoring, optical fibers can be used to detect structural parameters such as stress and strain, as well as environmental conditions responsible for corrosion, being moisture, pollutants, pH variations and metal ions. This type of sensing system consists of a transducer, a communication system and a signal detector/generator coupled with a signal processing and conditioning apparatus [53]. It can easily be integrated into the structure of the aircraft during the manufacturing process and provide real-time data about the condition of the structure over its entire lifecycle [80]. Fiber optic-based sensing can be conducted using two distinguishable configurations based on whether the optical fiber operates as both the transducer and the communication channel, as in the case of point-wise and quasi-distributed sensors, or solely as the communication channel as in the case for colorimetric sensors.

### 8.1. Point-Wise and Quasi-Distributed Fiber Optic Sensors

Point-wise and quasi-distributed optical fiber sensors are essentially optical fibers coated with an indicator film that interacts with target molecules or alters its structure in response to variation in environmental conditions. The interaction between the indicator molecules and the measurands result in a change in the refractive index of the cladding, which in turn alters the amplitude and/or the frequency of the optical signal propagating through the fiber core [13]. The most common fiber optic sensors used in SHM are backscattering fiber optics and fiber grating optical fibers. The main advantage of these types of sensors is that they can easily be integrated in distributed sensing systems that use a common source and common processing apparatus and provide multiple point measurements over long distances [80].

#### 8.1.1. Backscattering Optical Fiber-Based Sensors

**Figure 3:**
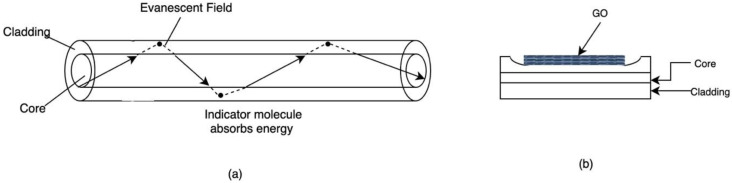
(**a**) Schematic of intrinsic optical fiber sensing mechanism. (**b**) Schematic of graphene coated Side Polished Fiber Reproduced with permission from Jun Zhang et al., 2014 Laser Phys. Lett. 11 035901.

Mendoza et al. proposed an optical fiber-based sensor that detects moisture and pH variation in aircraft lap joints [41]. In this approach, the sensing is conducted based on the interaction of the evanescent field between the fiber core and the cladding (Figure 3a). The fiber cladding is made of a moisture permeable polymer doped with indicator dye sensitive to the presence of water or changes in pH [41].

In the presence of water or when the pH varies beyond a pre-set range, the indicator dye alters the refractive index of the fiber cladding. As the light propagates in the fiber, the change in refractive index results in optical losses due the absorption of specific wavelength by the indicator molecules. The propagating optical signal is then transmitted to an array of photodetectors that converts it to electrical signal, which in turn, upon analysis, provide information about the type of measurand and its concentration [41]. Recent studies explored the feasibility of integrating thin film organic materials to fiber optics for chemical and bio- sensing. The interest stemmed from the various attributes of such materials like graphene in term of light-matter interaction, sensitivity, conductivity and chemical stability [81]. In this instance, the thin film is used as the fiber cladding with which the evanescent field interacts as the signal propagates through the fiber core [81]. Changes in the surrounding environment or the presence of analytes alter the structure of the sensing material and affects it refractive index [81]. Zhang et al. proposed a fiber optic temperature sensor using rGO as the sensing material. rGO was deposited on a side-polished optical fiber (SPF) and serves as the cladding in which the evanescent field propagates and interacts with the outside environment as shown in Figure 3b [81]. The used of rGO enhances the sensitivity of the temperature sensor due to strong optical absorption and high thermal conductivity. The proposed sensor is easy to fabricate and is characterized by high sensitivity, high precision and fast response [81].

#### 8.1.2. Fiber Grating Optical Fiber-Based Sensors

**Figure 4:**
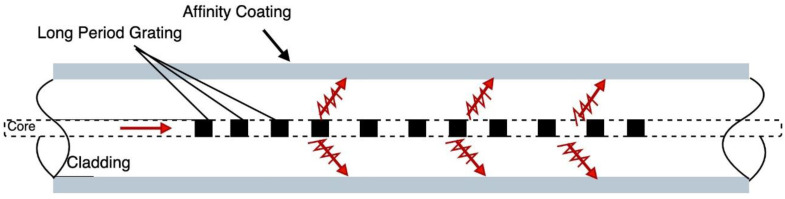
Schematic of long period grating optical fiber sensing mechanism. Reproduced with permission from Cooper et al., 2001 IEEE Autotestcon Proc. (2001).

Fiber grating sensors is another approach that has been widely used in structural health monitoring systems and can be potentially employed in corrosion monitoring in the aerospace industry. They are essentially periodic perturbations introduced to the refractive index of the fiber core, in such a way that specific wavelengths of the propagating optical signal are reflected, resulting in discrete spectral loss in the spectral response [82]. The fiber grating response depends heavily on the grating period, the fiber core and fiber cladding refractive indices, which makes it suitable for structural health monitoring applications [82]. The most commonly used type of fiber grating in SHM are Fiber Bragg Grating (FBG) and long-period grating (LPG). FBG, also known as short-period fiber grating, has a grating period in the hundreds of nanometers and commonly used for strain detection in structures. LPG, on the other hand, has a period ranging between 100 and 700 nm and is more suitable for chemical sensing [83]. Cooper et al. proposed an optical fiber-based chemical sensor to detect moisture and corrosion by-products such as Cu^2+^ in aircrafts lap joints, using an LPG coated with specially designed affinity film as shown in Figure 4 [82].

When the target species are absorbed by the affinity coating, the refractive index cladding changes, causing a shift in the wavelength of the reflected light. The shift in the spectral loss dip is correlated with concentration of the target species by comparing it to a spectral loss dip of a reference sensor [82].

Similar to previously described graphene coated SPFs, graphene-based materials can be used as the sensing components in Fiber Bragg Grating sensors to target specific chemical compounds and environmental parameters. In this context, Zhang et al. developed an ammonia gas sensor using microfiber FBGs coated with a layer of graphene oxide. This sensing scheme combines signal attenuation and Bragg wavelength shift induced by the interaction of GO and the evanescent field and the change in refractive index of GO respectively [84]. As in the case for graphene coated LPR, graphene oxide enhances the evanescent field in the microfiber and achieves high sensitivity and good repeatability at relatively small fiber diameters [84].

### 8.2. Optical Fiber Based Colorimetric Sensors—Phototransistors as a Detection Method

**Figure 5:**
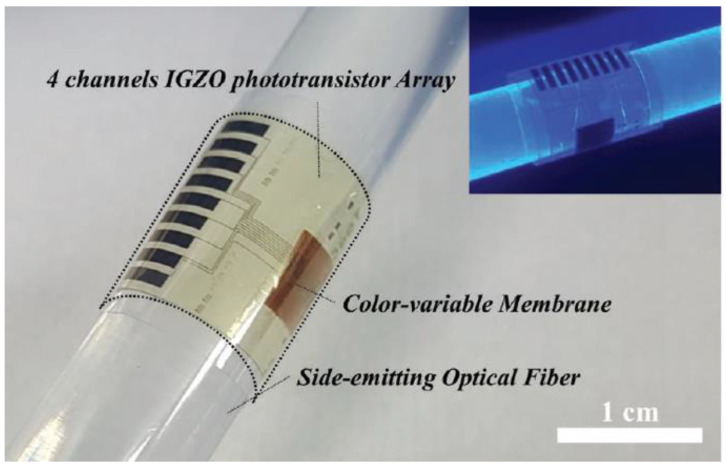
Multifunctional colorimetric light-transmittance-based durability diagnostic system. From Im et al., Adv. Mater 32(23) (2019).

Colorimetric sensing is an optical fiber-based scheme in which the sensing is conducted by an array of photodetectors, while the optical fiber operates as the communicating channel between the light source and the photodetectors. Im et al. proposed an optical fiber-based chemical sensor to measure chloride concentration and pH variations [85]. The system is essentially a distributed corrosion sensor based on colorimetric multifunctional photo-transmittance, that employs side emitting optical fibers coated with measurand-selective colour variable membranes and a flexible phototransistor array wrapped around the fiber as shown in Figure 5 [85]. The color variable membrane is designed to change colour when in contact with specific measurands and acts as a light filter for the light emitted by the fiber. The gradual change in the colour of the membrane is captured by the phototransistor array and converted to electrical signal providing information about the location and the concentration of the measurand [85].

The photo-sensing apparatus shown in Figure 5 is a flexible thin film phototransistor deposited on a polyimide (PI) flexible substrate and passivated with aluminium oxide (Al_2_O_3_) to prevent chemically induced stress [85]. Thin film transistors (TFT) are an attractive technology that have been successfully employed in biomedical and robotic sectors due to their small footprint, robustness, and low fabrication cost. Furthermore, TFT technology combined with advances in micro and nano-fabrication techniques is paving the way for organic semiconductor-based sensors that could be implemented in structural health monitoring applications such as corrosion monitoring.

**Figure 6:**
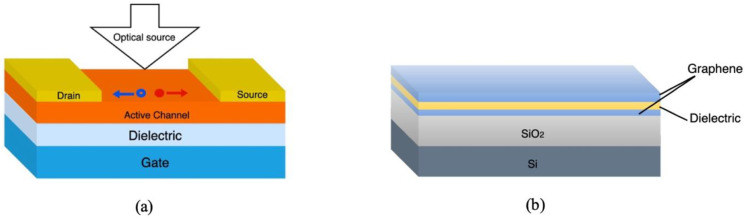
(**a**) Phototransistor lateral structure. (**b**) Schematic of graphene double layer heterostructure. Reproduced with permission from Liu et al., Nat. Nanotechnol. 9(4), 273–278 (2014).

The fiber optic based distributed sensing configurations previously described requires devices that convert the optical signal into electrical signal for processing purposes. This can be done by photodetectors such as phototransistors, which are active optoelectronic devices consisting of a semiconductor, source, drain and gate electrodes and dielectric material as shown in Figure 6a. The semiconducting channel generates electron-hole pairs in response to incident light. The source, drain and gate electrodes facilitate the charge flow from source to drain and the dielectric layer allows for the capacitive control of the charge carriers in the channel [86].

Graphene has emerged as the material of choice among organic semiconductors in a wide range of applications. In optoelectronics, graphene has a great potential due to its high optical sensitivity, large absorption bandwidth and excellent carrier transport [57]. However, single graphene layer-based photodetectors suffer from low absorption associated with the gapless nature of graphene and lack of mechanisms to multiply the photogenerated carriers [87]. Nonetheless, graphene is compatible with a variety of materials resulting in van der Wall heterostructures, can be chemically functionalized and can form hybrid heterostructures with nanoparticles, allowing for opening the bandgap and the enhancement the overall optical performance [87]. For instance, GO which is an oxidized form of graphene, has been extensively used in the fabrication of photodetectors due to its compatibility with numerous sensitizing materials such as nanoparticles and Transition-metal dichalcogenide (TMDs). GO-based hybrid and heterostructures have far better performance characteristics resulting from tunable bandgap, and high sensitivity [87]. In fact, GO bandgap engineering can be realised through fluorination (FGO), reduction (rGO) and hybridization using quantum dots or TMDs [87]. Although combining graphene with light sensitizing materials displays superior device responsivity, light absorption is restricted to the sensitizing materials used which limits the photodetection spectral range [88]. An interesting alternative was proposed by Chang-Hua Liu et al. where a phototransistor was fabricated using two graphene layers sandwiching a thin wide-bandgap dielectric layer as shown in Figure 6b [88].

In this configuration, the graphene layers act as light absorption channel and charge transport channel, while the charge separation is done by quantum tunneling. The heterostructure showed a high ON/OFF ratio and high ON current compared to other graphene-based devices [89]. In addition, the use of thin film semiconductors as a tunneling barrier allows for the fabrication of flexible and transparent photodetectors [89].

Fiber optic based distributed sensing systems have been widely deployed to implement structural health monitoring devices due to their advantages associated with remote and multiple point sensing, compactness, low transmission losses as well as high sensitivity. However, implementing fiber optic sensors in aircrafts health monitoring systems to detect corrosion environment and by-products, faces challenges related to coupling losses between the fibers and the photodetectors, and the difficulty of developing adequate sensing layers that do not affect the interaction between the target chemical species and the propagated light inside the fiber [80]. Optical fibers are also prone to bending and misalignment related issues caused during the installation and maintenance processes, as well as harsh environment conditions associated with avionic operations [80].

## 9. Thin Film Chemical Sensors

Chemical sensing methods have been used in structural health monitoring applications and can potentially be implemented in corrosion monitoring systems. In aircraft corrosion monitoring, the approach is based on sensing atmospheric corrosion parameters and corrosion rate of metallic structures, such as the presence of chloride or metal ions, using electrochemical probes or sensors such as ion selective electrodes (ISE), ion selective field effect transistors (ISFET), electrochemical capacitive sensors and pH sensors. Electrochemical probes and sensors are non-destructive, have good resolution and low power requirements [38].

### 9.1. Ion Selective Field Effect Transistor

**Figure 7:**
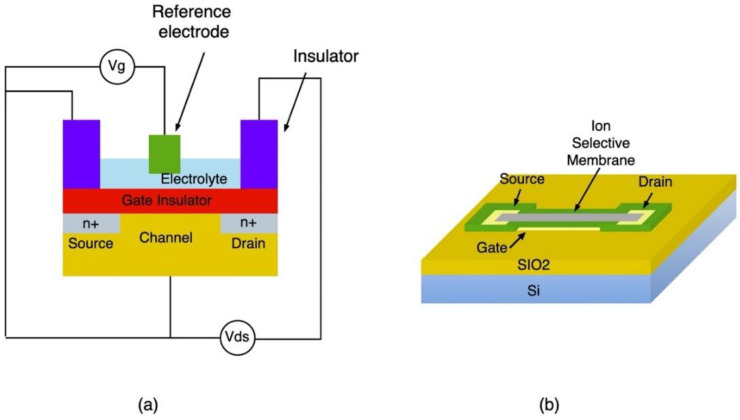
(**a**) Solution ISFET sensing system. Reproduced with permission from Akbari et al., Superlattice Microst. 130, 241–248 (2019). (**b**) Solid gate ISFET sensor structure. Reproduced with permission from Salvo et al., Sens. Actuators B xxx (2017).

Ion selective field effect transistors (ISFETs) are a class of potentiometric sensors that have been widely used in a variety of applications such as environmental monitoring, medical diagnostics and industrial process control [90]. They are essentially three-terminal semiconductor devices in which the current is controlled by an electric field. They are comprised of a gate channel, source and drain electrodes as well as a gate insulator. ISFETs are potentiometric sensors that produces electrical current in response to changes in ionic distribution. The sensing is conducted by coating the gate electrode with an ion-selective material such as ionophore mixtures or metal oxides, to which specific ions reversibly bind to and modulate the FET channel current and surface potential depending on the concentration of the ions [90]. Figure 7a illustrates the basic construction of a solution gate ISFET which include a reference electrode to provide steady reference potential and allow the FET to have a gate voltage [91]. Figure 7b shows the basic structure of a solid gate ISFET [44].

The state-of-the-art ISFET sensors use inorganic semiconductor materials, such as silicon, as the gate channel. However, scaling down such devices may be problematic as the resolution depends strongly on the channel size, carrier mobility as well as the capacitance between the FET channel and the ion binding sites [90]. Graphene based ISFETs can potentially overcome these challenges owing to graphene large surface to volume ratio and high carrier mobility [90]. Fakih et al. developed an electrochemical sensor array based on graphene ISFETs that measures the concentration of Cl^−^ and other ions, using ion selective membranes deposited on a graphene channel. The sensor array provides real-time and simultaneous measurement of multiple ions concentration in multi-ion electrolyte solution and exhibit high sensitivity and high resolution [90].

### 9.2. Ion Selective Probes

**Figure 8:**
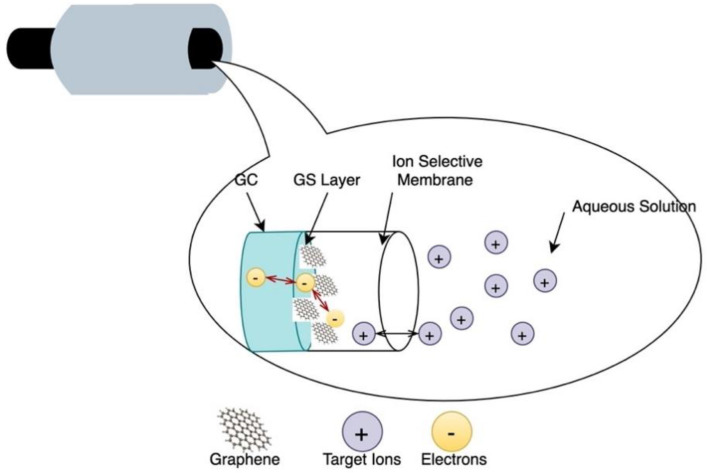
GC/graphene ion selective electrode structure. Reproduced with permission from Li et al., The Analyst. 137(3), 618–623 (2012).

Ion selective probes are another class of potentiometric sensing method to detect and measure the concentration of specific ions by measuring the voltage variations across two electrodes in the absence of current flow [92]. They have been widely used in clinical and environmental monitoring due to their robustness, selectivity over competing species, fast response and low-cost. Ion selective probes are integrated electrode systems consisting of two or three electrodes depending on their application. One of the electrodes is used as the working electrode (WE) and the others as the reference electrode (RE) and the counter electrode (CE) [38]. The electrodes are usually made from the same or different materials, but the working electrode is coated by an ion-selective material or membrane and acts as the transducer element, whereas the RE/CE maintains a constant potential and is insensitive to changes in the analyte composition [92]. The ions concentration measurement is carried out by bringing the electrodes in contact with the analytes. The interaction between the analytes and the ion selective membrane on the working electrode results in variations in the potential, which is correlated with the ion concentration [92]. There have been several attempts in the transition toward the use of organic materials such as graphene and its derivatives in the fabrication of chemical sensors due to their abundance, low-cost and competitive attributes in terms of sensitivity and fast response. For instance, Li et al. developed a graphene-based solid contact ion selective electrode (ISE) to measure the concentration of potassium [93]. The ISE is fabricated by depositing graphene sheets onto polished glassy carbon (GC) electrodes and coating the GC/graphene electrode with an ion-selective membrane as shown in Figure 8. The graphene-based ISE was tested using chronopotentiometry, potentiometric sensing and electrochemical impedance spectroscopy, where it was used as a working electrode [93]. The GC/graphene-based ion selective electrodes performance was comparable to carbon nanotubes (CNTs)- electrodes with improved long-term stability allowing for miniaturized and mass fabrication of ISE based sensors [93].

It can be inferred that the proposed graphene ion selective electrode may be used to measure the concentration of chemical compounds responsible for or resulting from corrosion events, such as chloride and metal oxides, by functionalizing graphene in such a way that it becomes selective towards those analytes [93].

### 9.3. Capacitive Sensors

**Figure 9:**
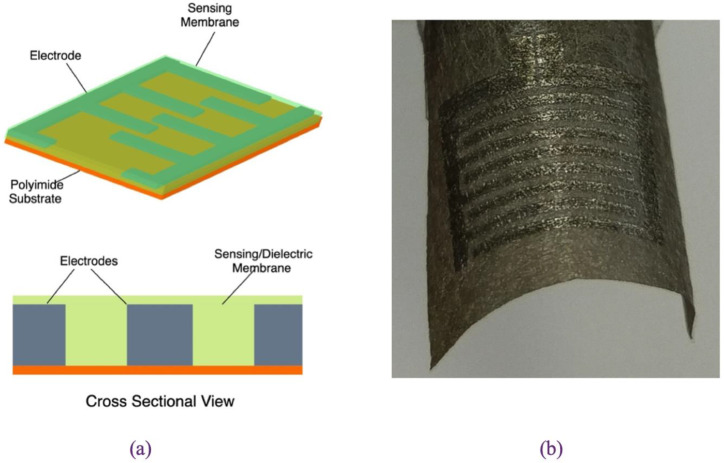
(**a**) Structure of IDC based chemical sensor. Reproduced with permission from Khan et al., Sensors. 16(5), (2016). (**b**) Flexible GO-based capacitive humidity sensor. Form R. Alrammouz, et al., Sensors & Actuators: B. Chemical 298 (2019).

Capacitive sensing is another sensing approach to detect the presence and measure the concentration of chemical species by measuring the changes in the capacitance between two or more conductors in a dielectric environment [40]. Capacitive sensors have been prevalent for a long time, mostly for humidity and pH sensing, and have recently attracted interest in chemical sensing application due to their robustness, high sensitivity, and compatibility with CMOS technology [40]. Chemical capacitive sensors have simple structures comprising of two interdigitated electrodes (IDEs) coated with an ion-selective dielectric layer as shown in Figure 9a [94]. When the sensitive layer absorbs analyte ions, its physical properties and dielectric constant change, changing thereby the generated potential.

Alrammouz et al. proposed a flexible capacitive humidity sensor using GO sheets as the sensing layer [73]. The sensor was fabricated by depositing a set of interdigitated electrodes on self-assembled GO on a paper-fibers as shown in Figure 9b. The porosity of self-assembled GO allows for the air flow monitoring from both directions which makes it suitable for monitoring of environmental factors. Strain tests showed that the bending angle had little to no effect on the sensitivity of the sensor which was comparable to resistive cellulosic paper-based sensors [73].

While ion selective and capacitive sensing approaches have been demonstrated to successfully detect specific ions, there is still room for improvement in terms of selectivity and resolution. The presence of other ions can interfere with the response of the sensors, as they can reversibly bind with the sensing membranes leading to unreliable measurements [90]. Furthermore, maintaining the sensitive membrane at a saturated state for long periods of time can be challenging, especially for polymer membranes, which compromise the long-term stability of the sensors [90].

### 9.4. pH Sensors

In corrosion monitoring, pH variations are an important parameter indicating corrosion events as well as its progress. Thus, monitoring pH would be beneficial in maintaining the safety and durability of aircraft structures. Over the years, several pH sensing approaches have been developed and the most common ones are based on paper strips and glass electrodes. However, the accuracy of these methods is limited by their fragility, low sensitivity and unsuitability for miniaturization [44]. Emerging pH sensing technologies are more focused on highly sensitive miniaturized robust devices that are able to provide in situ long-term pH monitoring in harsh environment, such as fiber optics, potentiometric sensors and ion selective field effect transistors (ISFET) [44]. Currently, ISFET is the most effective alternative to glass probes due to its small footprint, robustness and long-term durability [44]. As previously described, the performance of field effect transistors depends strongly on the sensitivity of the active channel as well as its responsivity. In this effect, numerous materials used in pH sensors have been reported in literature, such as aluminum oxide, poly(methacrylic acid) (PMAA), silicates, hydrogel and fluorescent dies, but their commercialization has been hindered due to their low sensitivity, degradation and difficulty of mass production [44]. Since graphene was successfully isolated from highly oriented pyrolytic graphite, it became a promising candidate in pH sensing applications due to its superior electrochemical responsivity compared to glassy carbon and CNTs electrodes, high reactivity to oxygen carrying groups like OH^−^, and efficient fluorescence quenching capabilities [44].

**Figure 10:**
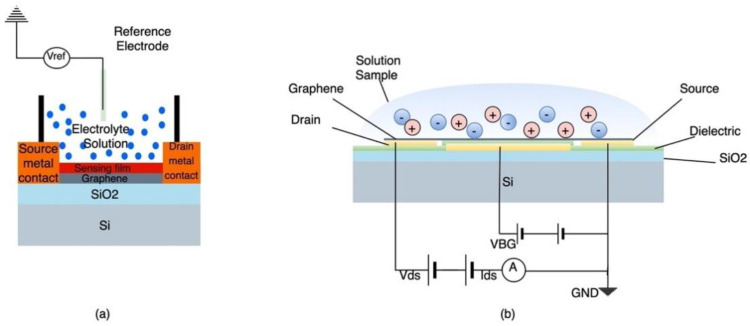
(**a**) Solution-gated ISFET structure. Reproduced with permission from Tarek et al., AIP Conf. Proc 1976, 020037,1–4, (2018). (**b**) Solid gated-ISFET based pH sensor. Reproduced with permission from Zhu et al., MEMS 2015, (2015).

Ion selective field effect transistors for pH sensing can be classified in two categories based on type of gate used- Solution-gated ISFETs and Solid gated-ISFET. Solution-gated ISFETs are field effect transistors resembling a three-electrode chemical cell, consisting of a conducting channel, a gate insulator, source and drain electrode and a reference electrode [44]. In this configuration, the electrolyte solution is in direct contact with the conducting channel and the gate voltage is directly applied through the reference electrode as shown in Figure 10a [44].

The use of the sensing film or the dielectric layer usually depends on the species to be measured. In the case of pH sensing, graphene is used as both the conducting channel and the sensing layer. Li et al. proposed a graphene-based solution gated-field effect transistor for pH sensing and compared the sensitivity of graphene monolayer (MG) to a few FLG. The graphene layers were grown by chemical vapour deposition (CVD) on a silicon oxide substrate [95]. Both configurations exhibited good sensitivity and resolution with FLG based sensor having faster response but lower resolution than the MG-based sensor after ions absorption [95].

Mailly-Giacchett et al. investigated the performance of flexible graphene based-ISFETs and the effect of fabrication-related residues on the electrical response of such pH sensors. The pH sensor was fabricated by growing a single layer of graphene by CVD on both silicon oxide and a poly(ethylene 2,6 naphthalenedicarboxylate) (PEN) flexible substrate [96]. Based on the experiment results, the sensing performance of the graphene grown on the flexible substrate was comparable to that grown on silicon oxide. In addition, the presence of a modest amount of fabrication related residues had little to no effect on the electrical response of the pH sensor [96].

Solid gated-ISFET structure is similar to the solution gated-FET but it is characterized by the absence of the reference electrode, which can be cumbersome in some applications. In this configuration, the dielectric layer is sandwiched between the gate electrode and the conductive layer, and the gate voltage is directly applied to the electrolyte through the gate dielectric as shown in Figure 10b [44]. Investigations into the influence of the gate dielectric characteristics on the performance of the sensors indicate that a material with a high dielectric constant allows for the dielectric layer thickness to be reduced, thereby lowering the leakage current while maintaining a high sensitivity [44]. For instance, Zhu et al. fabricated a graphene based solid gated-FET using a thin layer of HfO_2_ as gate dielectric. Usage of HfO_2_ of a high dielectric constant allowed for a significant reduction of the dielectric layer thickness to achieve high capacitance [97]. Since the sensitivity of the sensor is directly proportional to the capacitance of the dielectric, the pH sensor exhibits a large transconductance while operating at low voltages [97]. Another approach to improving the performance of solid gated-ISFETs, is the use of 3D graphene as the conducting channel, which was proposed by Ameri et al. The designed pH sensor makes use of graphene foam as the conducting layer coated with a sensitizing layer made of HfO_2_ [98]. The use of suspended 3D graphene configuration, combined with its large surface area, allows for thorough electrostatic gate control, which enhances the coupling between the electrolyte ions and the graphene layer [98]. The sensitivity of the device is also enhanced by the presence of binding sites for hydrogen ions provided by the HfO_2_ coating [98]. The proposed device demonstrated high pH sensitivity in high ionic strength media, offering the potential for real-time pH monitoring [98].

These experimental results highlight the versatility of graphene-based pH sensors in terms of ease of tunability, processability and compatibility with flexible printable devices. Each configuration offers unique advantages such as the use of reference electrodes in solution gated ISFETs which reduces the effects of solution variability, and its absence in solid gated ISFETs allows for device miniaturization and improves integration and microfabrication [44].

## 10. Wireless Sensors

Wireless sensing is a preferred alternative to fiber optic-based technologies in structural health monitoring applications, because it eliminates the need for active energy sources and active electronics at the sensing location and allows the energy and data to be transferred wirelessly. Eliminating the use electrical wires and batteries in some instances, enhances the stability and durability of the system in harsh environments. Wireless sensors are low-cost and small in size, and therefore, easily integrated with moving components and in inaccessible locations [13].

### 10.1. Surface Acoustic Wave Sensors

Surface Acoustic Waves (SAW) are mechanical waves confined to the surface of an elastic material and have an exponentially decaying amplitude through the material bulk [99]. The amplitude and frequency of this type of acoustic vibrations depend strongly on their environment, i.e., any change in the environmental parameters, such as temperature and pressure, affects the behaviour of SAW. The SAW technology has been extensively used in analytical processes in which target analytes induce changes in the environment, which alter the mechanical vibration properties such as frequency and amplitude. Consequently, SAW have been used as actuator to instigate a mechanical response is some materials such as piezoelectric crystals [99].

SAW-based sensors have attracted significant attention and are currently used as the primary components in a wide range of micro-sensing applications, due to their small footprint and ease of fabrication which only involves surface microfabrication techniques and modest spatial resolution [99]. They are compatible with CMOS technology and have a relatively low power consumption (~1 W, ~10 V_pp_) and high operational frequencies [99]. Their operating frequencies range from several hundred MHz to GHz allowing for the detection of small frequency shifts associated with diminutive mass loading [100].

**Figure 11:**
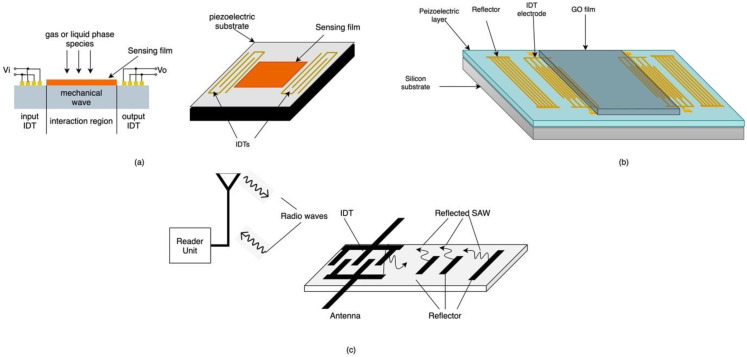
(**a**) Structure of Surface Acoustic Wave sensor. Reproduced with permission from Rocha et al., Sensors, 9(7), 5740-5769-2856, (2009). (**b**) GO film-based SAW humidity sensor. Reproduced with permission from Le et al., Microsyst. Nanoeng., 5(1), (2019). (**c**) SAW-based wireless sensor. Reproduced with permission from Aubert et al., ICEM., (2009).

SAW-based sensors consist of one or more interdigitated transducers (IDTs) deposited on an optically polished piezoelectric substrate [99]. An area of the substrate surface is functionalized to target specific chemical species, mechanical deformation or environmental parameters such as temperature and pressure as shown in Figure 11a.

The operation of SAW-based sensors is based on actuating the input IDT with an RF sinusoid or a windowed sinusoid, resulting in a mechanical wave traveling through the piezoelectric substrate at a specific frequency. In the presence of measurands, the sensing film on the substrate surface changes the frequency, amplitude and/or velocity of the propagating SAW, which is captured by the output IDT, reflected back to the input IDT and converted into electrical signal [101]. Piezoelectric materials are intrinsically affected by changes in temperature and strain, which eliminates the need of sensing materials for temperature and strain deformation monitoring [101]. Chemical and biosensors, on the other hand, require sensing materials that modify their structures as they selectively absorb chemical species and affect the surface of the substrate, which in turn alter the velocity of the propagating wave [101]. The most common materials used as sensing films in SAW-based sensors are polymers, especially in gas sensing applications, however they are not suitable for harsh environment applications due to their low thermal and mechanical stability [99]. Nanomaterials such as carbon nanotubes and metal oxide nanowires have had a better performance due to their high surface-to-volume ratio [99]. Recently, functionalized graphene has been reported promising in humidity sensing applications due to its large surface-to-volume ratio as well as its hydrophilicity and electrical insulation [74]. Le et al. proposed a SAW-based humidity sensor that uses GO as the sensing material as shown in Figure 11b. The device is fabricated by depositing a piezoelectric layer (AIN) on a silicon substrate and patterning it with gold to create the IDTs. A GO layer is then deposited on the AIN layer between the two sets of IDTs [74]. The humidity sensor operates at a frequency of 226 MHz and exhibits high sensitivity and high responsivity as well as short time recovery, good reversibility and excellent short-term repeatability [74].

Another advantage associated with SAW-based sensors is that they can operate in both wired and wireless mode. In wired mode, as the name suggests, the input IDT is connected to the reader or signal processing unit via electrical wires. In wireless mode, the transmission between the reader and the sensor is carried out via radio waves captured by antennas at the reader and at the input IDT as shown in Figure 11c [102].

### 10.2. Radio Frequency Identification Sensors

**Figure 12:**
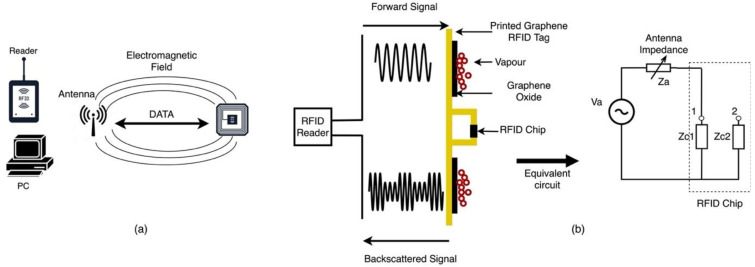
(**a**) RFID system components. From Wavelinx, RFID—Tags, Reader, Features and Working. Copyright 2020 Wavelinx Technologies Pvt Ltd. (**b**) Operating principle of the GO based printed graphene RFID sensor system. Reproduced with permission from Huang et al., Sci. Rep., 8(1), (2018).

Radio frequency identification (RFID) is a radio communication technology used to extract data electronically encoded in RFID tags or chips. Its operation principle is similar to that of barcodes except RFID readers do not need to be in proximity to the tag as the data is transmitted via radio waves [42]. RFID systems have three main components, a tag consisting of a microchip attached to an antenna and fixed to a plastic or a glass substrate, a reader made of a scanner, an antenna to receive and transmit data from the tag and a processor to record and process the reader’s data as shown in Figure 12a [42].

During the last decade, RFID has gained popularity as a form of identification technology and has been extensively used in a variety of industries such as agriculture, supply chain management as well as access control. More recently, RFID systems have been used in chemical and bio-sensing technologies namely environmental and health monitoring [42].

RFID-based sensing is achieved by depositing a sensing material either within the gap between the electrodes or by integrating the sensing material as part of the circuit components such as antenna or resonator [103]. Antennas and resonators are sensitive to their environment, and any changes induced by the sensing coating in the presence of target species will alter the signal transmitted to the reader [75]. Thin film materials have been the material of choice for RFID-based sensors due to their large surface-to-volume ratio, ease of functionalization and high thermal and electrical conductivity. Graphene derived materials, in particular, have been tested in RFID- devices and circuits such as interconnect, resonators and antennas [103]. For instance, Huang et al. proposed a wireless RFID- based relative humidity sensor by depositing a graphene oxide film on a graphene printed RFID antenna as shown in Figure 12b [75]. When the reader transmits an electromagnetic signal to the tag, the antenna uses some of the signal energy to power the microchip on the tag, which in turn, generates a modulated backscattered signal by varying its impedance. The RFID antenna is designed to match its impedance to that of the tag chip, and since the antenna is sensitive to changes in the environment due to proximity effects, any changes in the GO film in the presence of humidity induce a change in the antenna’s impedance, leading to changes in the backscattered signal phase, which is then transmitted to the reader [75].

It is worth noting that the proposed device is a printable wireless battery-free RFID-based sensors that rely on the dielectric properties of GO to detect and measure the relative humidity of the surrounding environment. The sensing scheme relies on changes in backscattered signal phase rather than resonance frequency, which significantly speeds up and simplifies the monitoring process [75].

Although wireless sensors seem promising, their implementation in avionic structural health monitoring faces numerous challenges associated with the harsh operational environments, including a broad range of temperature, pressure, liquids, as well as mechanical loading, vibrations and associated noise [104]. As in the case of most wireless systems, the reliability of wireless sensors could be affected by electromagnetic interference due to the large number of devices communicating between each other during flight operations. Furthermore, small sensors require high frequency antennas to transmit data at higher rates, which reduces the transmission range, in addition to the complexity of small sensors fabrication process which increases the cost [104].

### 10.3. Sensor Nodes

**Figure 13:**
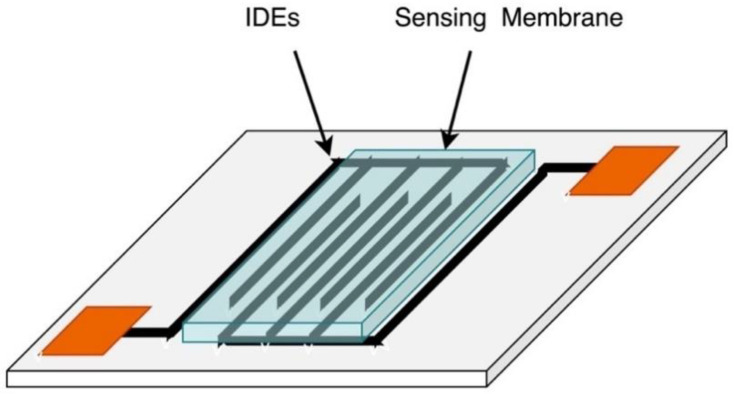
Schematic of an IDEs chip with PANI/CNTs conductive coating. Reproduced with permission from Rinaldi et al., Int. J. Aerospace Eng., 2012,1–11, (2012).

In the aircraft industry, monitoring corrosion is crucial in ensuring the durability and safety of aircrafts, which requires arrays of sensors that track various chemical species responsible and indicative of corrosion damage. Tracking the various types of chemical species on the same structure can quickly become complex and costly as each sensor needs its own circuitry to relay sensor data. Wireless sensing networks is an attractive concept of distributive real-time monitoring, where a number of sensor nodes are used to monitor different chemical species and transmit their data to a central data acquisition unit [105]. Sensor nodes are simple transducers with sensing, networking and in some cases, processing capabilities, integrated along with other sensors in a sensing interface, all connected to a controller board that manages data acquisitions from the various nodes and performs processing operations [105]. The concept of multi-parameter integrated sensors was investigated by Rinaldi et al., where a distributed corrosion sensing network was proposed to detect hydrogen, relative humidity and temperature variations [54]. The system includes polyaniline/carbon nanotubes-based sensors for hydrogen gas and humidity detection [54]. Polyaniline (PANI) is an intrinsically conductive polymer characterised by high conductivity, low density, environmental stability and ease of processability. Combining carbon nanotubes (CNTs) with PANI enhances the conductivity of the semiconductor by improving the charge carrier mechanism in PANI [54]. In this configuration, PANI/CNTs composite material was deposited on simple sensing chip made of interdigitated electrodes as shown in Figure 13 [54].

The sensor exhibits excellent sensitivity to humidity compared to commercially available sensors [54]. However, its response to variation in hydrogen concentration was limited by hydrogen bonding sites in the PANI/CNTs material or the presence of residual moisture as a result of the fabrication process [54]. Nonetheless, the concept presented is indicative of the feasibility of such networks which features the advantages of sensing nodes in decreasing the complexity of distributed sensing systems combined with the benefits of using organic semiconductors.

Sensor nodes is a monolithic integration approach that incorporate sensors targeting different analytes in the same node. It is a novel approach that can be useful in aircraft corrosion monitoring, where several sensors targeting the corrosion factors outlined in Figure 2, are encapsulated in the same node and placed in critical locations of the aircraft structure. The sensor nodes can have a common data acquisition and processing unit and the connection can wired or wireless or both using wireless sensors like SAW and RFID. This configuration allows for continuous monitoring of corrosion environment, by-products and progress while decreasing the complexity of the sensing network and the associated installation and maintenance costs.

## 11. Conclusions and Outlook

Corrosion of metallic aircraft structures continues to be a major concern of aircraft structural integrity. Under the current “find and fix” maintenance approach, corrosion detection involves visual inspection as well as sensing techniques focused on structural damage caused by corrosion in terms of mass loss, coating degradation and structural defects. These approaches come with substantial drawbacks associated with maintenance scheduling restrictions, the inability to distinguish between cracks and corrosion and the different forms of corrosions and the fact that the early stages of corrosion can often go undetected. These drawbacks can lead to undesirable consequences, such as increased aircraft downtimes and maintenance and repair costs, and catastrophic failure in worst case scenarios. To address the needs for enhanced aircraft availability and reduced maintenance cost, there has been a shift within aircraft maintenance community towards corrosion- and crack- condition based aircraft management that enables early corrosion detection, and continued monitoring and integration of corrosion rates into the aircraft structural integrity program. The new management regimes are expected to reduce substantially costs through more efficient maintenance and proactive corrosion preventative measures. The core to this new management regimes is an integrated management system consisting of reliable and durable corrosion sensors and accurate methods to predict corrosion conditions.

This review covers the main corrosion forms of typical aircraft structures, and their mechanisms and impact on aircraft structural integrity. It demonstrates the diversity and complexity of corrosion, as well as challenges in predicting corrosion conditions. In order to achieve reliable corrosion inspection, the review discusses the three categories of corrosion index, including physical, chemical and environmental corrosion index. The review provides detailed descriptions of the existing sensing technologies for detection of physical changes such as thickness change of a structure, chemical by-products from chemical reactions of corrosion, as well as corrosion-conducive environmental conditions for corrosion severity and rate determination, including temperature, humidity and time of wetness among other factors. The state-of-art of the three categories of corrosion sensors is given in this review with regards to their limitations, false-positive reading rates and durability, pointing to the need for high-sensitivity and high-fidelity integrated chemical sensors to enhance corrosion detection and monitoring. The emerging graphene-based corrosion sensing approaches covered in this review offer an effective alternative to the currently employed techniques due to their many advantages, such as real-time monitoring of corrosive environment, and detection capabilities of early stages of corrosion events. These emerging technologies can also monitor key parameters responsible and indicative of corrosion, namely environmental factors and corrosion byproducts. Such parameters offer valuable information for corrosion mechanisms as well as for corrosion prediction, prevention, and maintenance. The alternative approaches rely on small footprint sensors that can easily be integrated into aircraft structures for multiple-point remote and real-time measurements. The use of novel organic thin film materials enhances the sensitivity, selectivity and reliability of the sensors as their high resistance to contamination reduces the likelihood of false positive readings. Furthermore, the suggested approaches use graphene derived materials as the sensing layers, which possess exceptional electronic, chemical, and optical properties. Table 3 summarizes and compares the performance of the sensors discussed throughout the review in terms of sensitivity, detection limit and response time. These performance parameters illustrate the advantages of using graphene as the sensing layer in relation to lower detection limits and higher sensitivity. However, using graphene derived material in chemical sensing is still at the experimental stage and requires further improvements toward selectivity, specificity, and long-term stability. The major challenges reside in the complexity of functionalization process which require control of process parameters at the atomic level to ensure the reliability and repeatability of the sensors as well as their selectivity and specificity [106,107]. In fact, the crystallographic orientation, density and amount of the functionalization agent have an impact on the surface properties and the long-term stability of the functionalized materials [106]. Graphene and graphene derived material are highly sensitive to environmental factors such as humidity as well as incident light leading performance discrepancies and degradation, environmental contamination and photodegradation [106,107]. Innovations in functionalization and fabrication processes are crucial to ensure the stability and repeatability of graphene-based corrosion sensors and facilitate large scale production and commercialization.

Nonetheless, advancements in chemical functionalization and patterning techniques allow for the transition toward miniaturized and monolithic sensing systems capable of monitoring multiple parameters simultaneously on a continuous basis. Furthermore, implementation of graphene derived materials in the emerging corrosion sensing techniques as presented in this review offers a wealth of possibilities for the realization of efficient and reliable real-time corrosion sensors for aircraft structural health monitoring while reducing maintenance and repair costs and maintaining the durability, sustainability, and safety of the fleets.

## Figures and Tables

**Table 1:** Corrosion forms, definition, and influencing factors (Revised based on [9]).

Form of Corrosion	Definitions	When to Consider	Influencing Factors
Uniform (general)	A form of corrosion that occurs uniformly over the entire exposed surface of a metal.	Susceptible alloys	pH, temperature, ionic pollutants, aeration, uniformity and adherence of scale surface finish
Pitting	A form of localized corrosion that occurs when a corrosive environment medium attacks a metal at specific points and results in deep cavities in the metal. This is one of the most widespread and detrimental forms of electromechanical deterioration	Aluminum, stainless steel and other alloys in aircraft structures, in particular Al 7000 series	Surface finish including flaws and cracks, stagnant fluids, ionic potential of the electrolytes
Crevice	A form of corrosion that occurs when an electrolyte becomes trapped and stagnant, in particular locations such as joints, corners, and under debris.	Joints, corners, and where debris may accumulate	Stagnation of pollutants due to poor fluid flow results in excessive ions available to precipitate corrosion reactions
Galvanic	A form of corrosion resulting from the formation of a galvanic cell by the galvanic coupling of dissimilar metals (metals having different electrical potentials), which are exposed to an electrolyte.	Two dissimilar metals in direct contact or separated but in electrical contact, such as carbon/Al, or aluminum joints with steel bolts	Dissimilar corrosion potentials between adjacent materials, ratio of exposed anodic and cathodic materials, pH, aeration, and temperature
Erosion Corrosion	The increased rate of deterioration and loss of a material due to the combined effects of corrosion and the repeated motion of the surrounding environment such as repeated impacts from hard particles.	Moving corrosive environment or erosive hard particles or liquid.	Turbulent flow, fluid velocity, density, angle of impact, cavitation
Intergranular	A form of corrosion that attacks grain boundaries in materials. It may occur as a result of a galvanic couple between differing phases within a material. It is also associated with the propagation of pitting and exfoliation corrosion	Susceptible alloy/heat treatments	Alloying content impurities within the metal, heat treatment, welding effects, 2nd phase precipitation products on grain boundaries
Stress corrosion cracking	A cracking process involving the combined factors of corrosive environment and a sustained tensile stress.	Static stresses	Tensile stress (applied mechanical or thermal, residual),
Fretting Corrosion	A form of corrosion caused by repetitive friction between two surfaces in sliding motion with respect to each other while exposed to a corrosive environment.	Small relative movement between two metals typically caused by vibration or repeated thermal expansion and contraction cycles	Vibratory movement between adjacent loaded components
Corrosion fatigue	The failure of a material due to the combined effects of corrosion and fatigue (cyclic stressing).	Cyclic stresses	Cyclic load
Hydrogen damage	Any deterioration of a material due to the presence of hydrogen, whether in the surrounding environment or internal to the material.	Hydrogen generation during processing or in service directly or indirectly, in particular, landing gear and engine components	High temperature moist environments, electrolysis, hydrogen evolved from other corrosion mechanisms
Exfoliation	A form of corrosion that occurs in bands on the interior of the metal that is parallel to the metal’s surface. This consequently forms corrosion products that cause separation of the metal into layers. It is often considered a form of intergranular corrosion that attacks metals that have been mechanically treated to form elongated grain structures in one direction.	Rolled and extruded alloys susceptible to intergranular corrosion	Similar to intergranular
Filiform corrosion	A form of corrosion that exists under organic and metallic coatings on metals, blistering the coating, and is characterized by hairline resemblance.	Thin permeable organic coatings on metals	Water permeable organic coatings or edges of alloys coated with metallic coatings exposed to high humidity

**Table 2:** Existing Corrosion Sensor Types.

Corrosion Factors	Sensors	Description	Pros and Cons
Physical	Visual/enhanced visual inspection [32] Eddy current [32] Radiography [32] Thermography [32] Electrical/inductive Resistance based mass loss sensors [33] Ultrasonic Sensors [13] Corrosion Potential-electric conductivity [34]. Linear Polarization Resistance [33] Galvanic corrosion sensor [33,35] Electrochemical Impedance Spectroscopy [36] Electrochemical noise [37] Acoustic emission [36]	Inspection of surface, intergranular, exfoliation and pitting corrosion. Measurements of mass loss, defects, cracks and coating degradation due to corrosion. Measurement of change in resistance, galvanic current, potential, impedance and acoustic emissions due to the presence of corrosion and the resulting mass loss.	Low-cost and broad applications. Mature technologies. Online or offline measurements. ○ May not be able to distinguish corrosion from cracks. ○ May be applicable to uniform corrosion only. ○ Difficult to implement in distributed systems. ○ Difficult to detect corrosion in hard-to-reach locations.
Environmental	Ion Selective Sensors (ISFET) [38] Electrochemical/biological sensors [38,39] Capacitive sensors [40] Fiber Optics [13] Photosensors [41] Surface Acoustic Waves (SAW) [13] Radio Frequency Identification (RFID) [42] Hydrogen probes [43]	Monitoring of environmental factors responsible for corrosion events such as moisture, temperature and pollutants. Monitoring of environmental factors through change in electrical, optical and acoustic responses of the sensors.	Real time on-board monitoring. Easy to integrate into moving components and hard-to-access locations. May be integrated into distributed networks. Possibility of wireless signal transfer. ○ Prone to electromagnetic interference. ○ May be affected by exposure to harsh environments. ○ Prone to signal noise.
Chemical	ISFET [38] Electrochemical sensors [38] Capacitive sensors [40] Fiber Optics [13] Photosensors [41] pH sensors [41,44] Color-indicating-paints [45].	Detection of by-products of corrosion such as, pH variation and metal ions. Change in electrical and optical response of sensors is induced by the presence of corrosion by-products.	Real-time on-board monitoring. Easy implementation in distributed systems. Versatile, compact and sensitive for chemical detection. High selectivity toward specific chemical species. ○ Lack of long-term stability and vulnerability to noise. ○ Selectivity issues in the presence of other chemical species.

**Table 3:** Graphene-based sensors performance comparison.

Sensor	Sensor Type	Parameter	Sensitivity	Detection Limit	Response Time/Speed	Ref.
Optical	Side Polished Fiber (SPF)	Temperature	0.134 dB/°C	0.9 °C	0.0228 °C s^−1^	[81]
	FBG	NH_3_	0.003–0.008 dB/ppm	Not provided	10 s	[84,108]
Potentio-metric	ISFET	Cl^−^, Na^+^, SO_4_^2−^, K^+^, NH_4_^+^	2 × 10^−3^ log	10^−5^ M	Not provided	[90]
		pH	34.5–57.5% (MG) 4.75% (FLG) 22 mV/pH (CVD) 71 ± 7 mV/pH (HfO_2_)	Not provided	<4 s	[95,96,98]
	ISE	K^+^	59.2 mV/decade aK^+^	10^−5^ M	<10 s	[93]
Capacitive	Capacitive Chemical Sensor	Moisture	5.65 fF/%RH	Not provided	Not provided	[73]
Frequency	SAW	RH	25.3 kHz/%RH	Not provided	<10 s	[74]
Phase	RFID	RH	0.5°/1%RH	Not provided	Not provided	[75]

## Data Availability

Not Applicable.
